# Clinical Prediction Nomograms to Assess Overall Survival and Disease-Specific Survival of Patients with Salivary Gland Adenoid Cystic Carcinoma

**DOI:** 10.1155/2022/7894523

**Published:** 2022-08-29

**Authors:** Hong-shi Cai, Shuo-jin Huang, Jian-feng Liang, Yue Zhu, Jin-song Hou

**Affiliations:** ^1^Department of Oral and Maxillofacial Surgery, Hospital of Stomatology, Guanghua School of Stomatology, Sun Yat-sen University, Guangzhou, 510055 Guangdong, China; ^2^Guangdong Provincial Key Laboratory of Stomatology, Guangzhou 510080 Guangdong, China

## Abstract

**Aim:**

Salivary gland adenoid cystic carcinoma (SACC) is the second highest incidence of malignant salivary gland tumor. The purpose of this study was to establish nomograms combined with SACC patients based on the Surveillance, Epidemiology, and End Results (SEER) database.

**Methods:**

Patients with SACC were included in the SEER∗Stat Database from 2004 to 2016. The least absolute shrinkage and selection operator (LASSO) Cox regression analysis was applied to filter potential prognostic clinical variables. Multivariate analysis from the Cox proportional hazards model was performed to determine the independent prognostic factors on overall survival (OS) and disease-specific survival (DSS), applied to develop nomograms. The Schönfeld residual test verified the proportional hazard assumption. The discrimination and consistency of nomograms was assessed and validated according to concordance index (*C*-index), receiver operating characteristic (ROC) curves, and calibration curves using an internal 1,000 times bootstrap resampling. The nomogram's net clinical benefit was assessed through decision curve analysis (DCA).

**Results:**

A total of 658 patients with SACC were included. Age, T stage, N stage, M stage, histologic grade, and surgery were independent prognostic factors for OS and DSS. Based on these independent prognostic factors, nomograms were developed to predict 3-, 5-, and 10-year OS and DSS. In the validation of 1,000 times bootstrap resampling, the *C*-index and ROC curves had good discriminatory ability. The calibration curves indicated excellent consistency between the predicted and actual survival results in the nomograms. The DCA curves demonstrated that the nomograms had good clinical benefit and were superior to the TNM stage and other variables.

**Conclusions:**

Two nomograms developed in this study precisely predicted the 3-, 5-, and 10-year OS and DSS rates of patients with SACC in accordance with independent prognostic factors, and their clinical value is better than TNM staging, providing a prognostic reference for other SACC patients.

## 1. Introduction

Salivary gland adenoid cystic carcinoma (SACC), which is the second highest incidence malignant salivary gland neoplasm characterized by strong invasiveness, local recurrences, perineural invasion (PNI), and hematogenous metastasis [1, 2], comprises approximately 10% of salivary gland tumors, and about 1% of all the head and neck malignancies [3–5]. With the primary site of SACC, it most commonly arises from small salivary glands of the oral cavity (palate, tongue, buccal mucosa, and lip) [2, 6] and then from major glands, with the submandibular gland and parotid gland most commonly affected (53.3% and 46.7%, respectively) [7].

Generally, SACC has an indolent yet unremitting clinical disease course with a low incidence of regional lymph node metastases. In sharp contrast, locoregional recurrence and distant metastasis are quite common, sometimes even years after surgical resection of the primary tumor. One of the reasons is that it spreads to the perineural with occult extension exceed surgical margin [8]. Hematogenous metastasis occurs in about 20–50% of patients, and the most frequently metastatic site are the lungs, followed by the bone, brain, and liver [9]. Therefore, SACC is considered a high-grade and unpredictable tumor with a poor long-term prognosis [10]. Radical surgical resection was used as a mainstay treatment for patients with SACC. However, it is difficult to achieve clear surgical margins for the strong invasiveness and high probability of PNI of tumor. Consequently, the combination of postoperative radiotherapy with surgery was used to achieve an improved locoregional control [11–13].

With the ability to integrate diverse prognostic and determinant variables, nomogram has been widely used as an evidence-based and practical means to define the prognostic factors and evaluate the prognosis of many types of cancer [14, 15]. Several studies have shown that nomograms are scientific and precise, which can make it an alternative to the traditional TNM staging system [16, 17]. To the best of our knowledge, large-scale researches on the precise risk evaluation of SACC are limited, and to date, a nomogram for patients with SACC has not yet been developed. Therefore, in order to gain a better understanding of SACC and optimize individualized prognosis assessment, treatment, and follow-up, we developed a nomogram to predict the 3-, 5-, and 10-year overall survival (OS) rate and disease-specific survival (DSS) rate of SACC patients by extracting data from the National Cancer Institute's Surveillance, Epidemiology, and End Results (SEER) database.

## 2. Materials and Methods

### 2.1. Data Source

Using SEER∗Stat software (version 8.3.8) to extract data of patients with salivary gland adenoid cystic carcinoma (SACC) from SEER∗Stat Database: Incidence-SEER 18 Regs Custom Data (with additional treatment fields), Nov 2018 Sub (1975–2016, varying), National Cancer Institute, Division of Cancer Control and Population Sciences (DCCPS), SEER Program, based on the November 2018 Submission. No approval by the institutional review board was sought since SEER is a public database.

### 2.2. Data Extraction

Variables for this study included age, gender, race, marital status, primary site of tumor, AJCC Stage (7th edition), TNM stage, histologic grade, surgery, radiation, chemotherapy, and neck dissection. We identified all patients with SACC between 2004 and 2016 from SEER∗Stat Database. The inclusion criteria were as follows: (1) confirmed histologic type of adenoid cystic carcinoma, (2) sites limited to salivary glands, (3) the first and only primary tumor, and (4) known causes of death and complete follow-up data. The exclusion criteria were as follows: (1) unknown grade, surgery, radiation, chemotherapy, and race; (2) by incomplete AJCC 7th Edition TNM Staging System; and (3) survival time ≤ 1 months.

### 2.3. Statistic Methods

Overall survival (OS) and disease-specific survival (DSS) were the identified endpoints. OS was defined as the period between the primary surgical treatment of the SACC and the time of death from any cause, or the last follow-up. DSS was defined as the period between the date of surgery and death resulting from SACC, or the last follow-up.

Twelve variables included in this study were: age, gender, race, marital status, primary site of tumor, AJCC Stage (7th edition), TNM stage, histologic grade, surgery, radiation, chemotherapy, and neck dissection. To reduce data dimensionality and filter predictor variables to minimize the risk of overfitting, the least absolute shrinkage and selection operator (LASSO) Cox regression analysis was utilized to construct a prognostic model [18]. Furthermore, multivariate analysis from the Cox proportional hazards model was used to determine the independent prognostic factors on OS and DSS of patients with SACC. The proportional hazard (pH) assumption was verified through the Schönfeld residual test. Then, the nomograms associated with 3- and 5-year OS and DSS were established by incorporating the independent prognostic factors. 1,000 times bootstrap resampling validated internally the performance of the nomogram. In order to describe the discrimination between predicted probability and actual observations of the nomogram, Harrell's concordance index (*C*-index) and the area under the receiver operating characteristic (ROC) curve (AUC) ranging from 0.5 to 1.0 were measured. The value of the AUC is equal to the value of *C*-index. Generally, a value of 0.50-0.70 means low prediction accuracy, 0.71-0.90 means medium prediction accuracy, and a value higher than 0.90 means high prediction accuracy. Calibration curves were used to evaluate the consistency between the predicted and observed results. Moreover, we assessed the potential clinical benefit of nomograms through decision curve analysis (DCA).

A two-sided *P* value < 0.05 was considered statistically significant. The study was conducted by using R version 4.0.5 (https://www.r-project.org/).

## 3. Results and Discussion

### 3.1. Search Results

**Figure 1:**
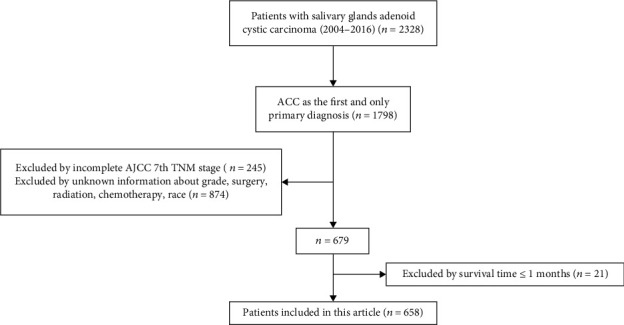
The flowchart displaying the selection process of patients with SACC from the SEER∗Stat Database. SACC: salivary gland adenoid cystic carcinoma; SEER: Surveillance, Epidemiology, and End Results.

We identified all 2328 patients with SACC between 2004 and 2016 from SEER∗Stat Database. Among them, 1798 patients meet the inclusion criterion: SACC as the first and only primary diagnosis. Then, 245 patients were excluded by incomplete AJCC 7th TNM stage; 874 patients were excluded by unknown information about grade, surgery, radiation, chemotherapy, and race; 21 patients were excluded by survival time less than 2 months. Finally, a total of 658 patients with SACC were included in this article (Figure 1).

### 3.2. Clinicopathologic Characteristics and Survival Outcomes

The median age was 56 years (ranging from 11 to 93 years). There were 379 (57.6%) female and 279 (42.4%) male patients. Among them, the primary sites most occurred in the parotid gland (30.2%), followed by the palate (15.8%). In the AJCC 7th TNM staging, stages I, II, III, IVA, IVB, and IVC accounted for 26.0%, 22.8%, 18.1%, 21.1%, 4.45%, and 7.6% of cases, respectively. As for histologic grade, there were 161 (24.5%) cases with low-grade transformation, 309 (46.9%) cases with intermediate-grade transformation, 113 (17.2%) cases with high-grade transformation, and 75 (11.4%) cases with high-grade transformation. In terms of receiving treatment, 629 (95.6%) patients underwent surgery and 363 (55.2%) received neck dissection. Additionally, 489 (74.3%) and 72 (10.9%) patients received radiation treatment and chemotherapy, respectively. Other detailed clinicopathologic characteristics are listed in Table 1.

Overall, the 658 included patients were followed for a mean of 44 months (range, 2-154 months). The 3-, 5-, and 10-year OS rates were 85.4%, 80.9%, and 76.7%, respectively, and the corresponding DSS rates were 87.7%, 83.7%, and 80.9%, respectively. The OS and DSS rates of all patients with SACC in terms of different clinical features are shown in Table 1.

### 3.3. Construction of the Prognostic Nomograms for OS and DSS

**Figure 2:**
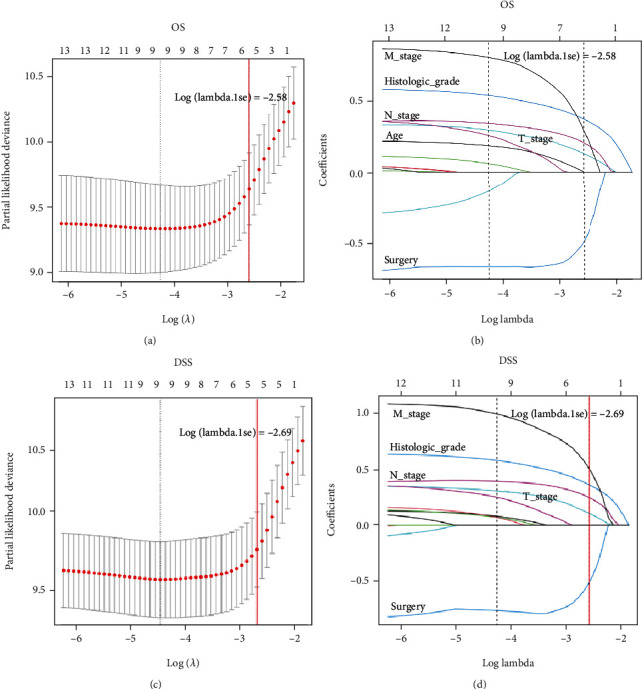
Used LASSO COX regression model to filter predictor variables for OS and DSS. (a) Selection of lambda.1se identified 6 variables for OS in LASSO Cox analysis. (b) LASSO coefficient profiles of 13 variables for OS. (c) Selection of lambda.1se identified 5 variables for DSS in LASSO Cox analysis. (d) LASSO coefficient profiles of 13 variables for DSS. Notes—1: age, 2: gender, 3: primary site of tumor, 4: histologic grade, 5: T stage, 6: N stage, 7: M stage, 8: race, 9: marital status, 10: surgery, 11: radiation, 12: chemotherapy, and 13: neck dissection.

**Figure 3:**
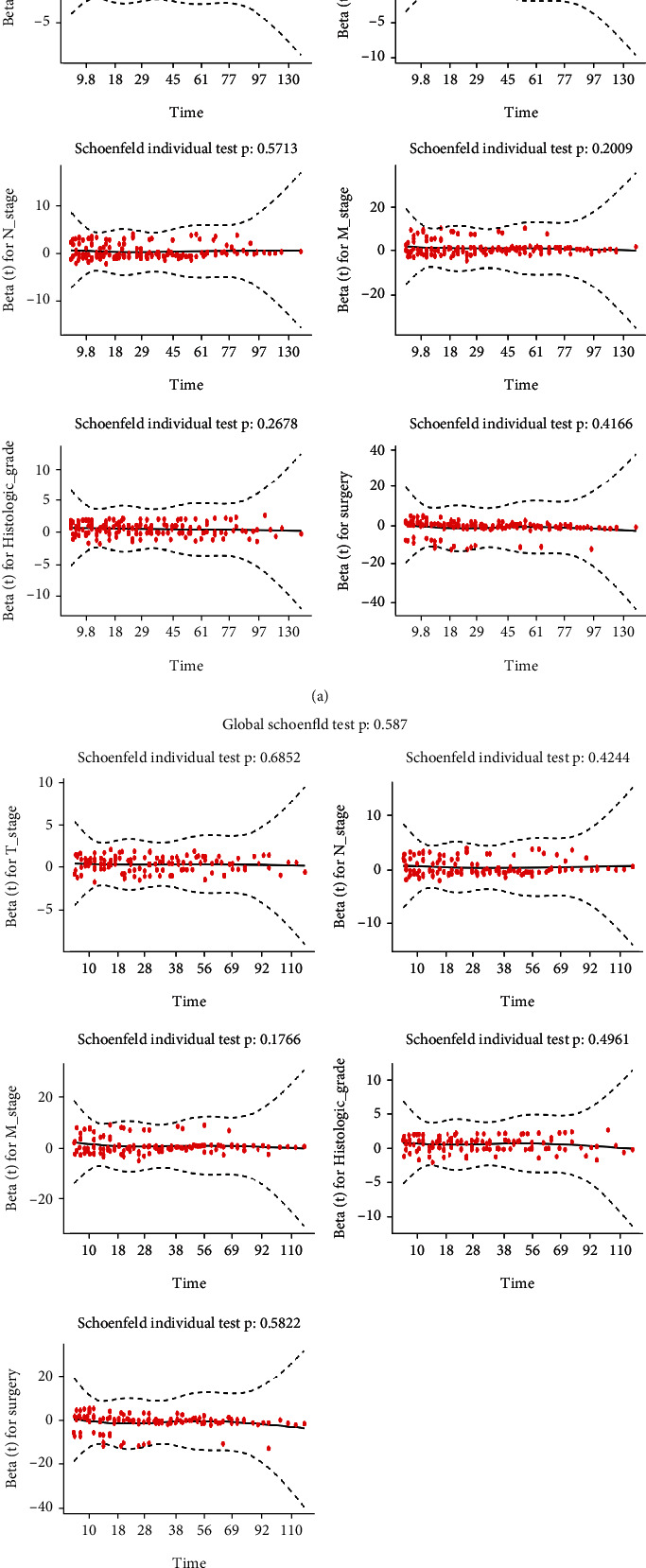
The pH assumption that met the cox proportional hazards model of OS (a) and DSS (b) verified by the Schönfeld residual test.

**Figure 4:**
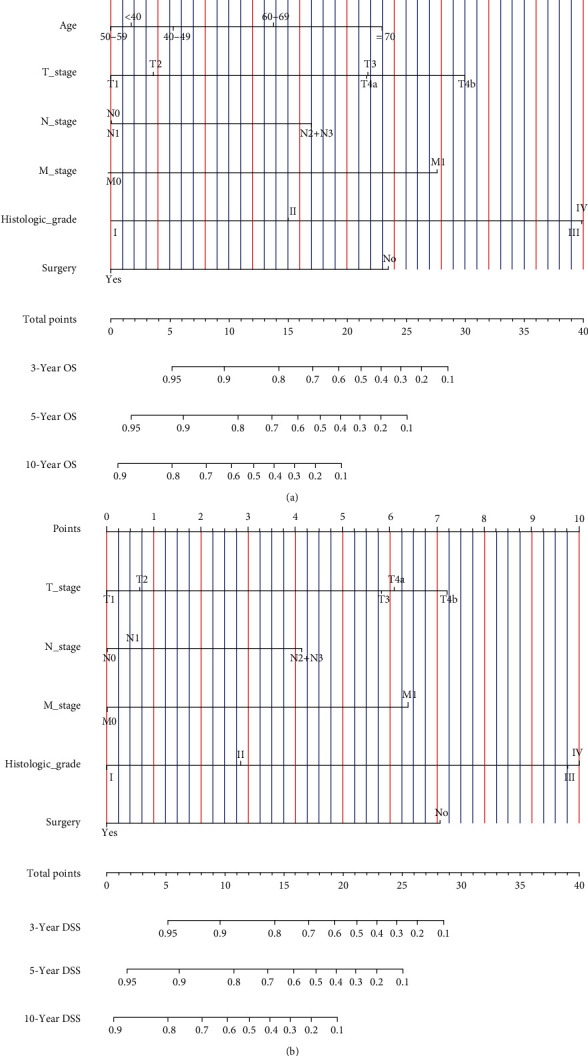
Construction of nomograms. (a) Nomogram predicting 3-, 5-, and 10-year OS in patients with SACC from SEER∗Stat Database between 2004 and 2016. (b) Nomogram predicting 3-, 5-, and 10-year DSS in patients with SACC from SEER∗Stat Database between 2004 and 2016.

Lambda.1se refers to the lambda value of the simplest model obtained within a standard deviation of lambda.min. In order to construct more streamlined predictive nomograms and prevent the nomogram from over-fitting, this study used the variables corresponding to lambda.lse to construct the nomogram. In total, 13 variables were simplified to 6 predictor variables (age, T stage, N stage, M stage, histologic grade, and surgery) for OS and 5 predictor variables (T stage, N stage, M stage, histologic grade, and surgery) for DSS that displayed in LASSO Cox regression analysis [19] (Figures 2(a)–2(d), Table S1). Next, we performed the multivariate Cox proportional hazard model to verify whether these 6 predictor variables were independent prognostic factors, age (*P* < 0.001), T stage (*P* < 0.001), N stage (*P* < 0.001), M stage (*P* < 0.001), histologic grade (*P* < 0.001), and surgery (*P* < 0.001) were independently and significantly associated with OS, while laterality, T stage (*P* < 0.001), N stage (*P* < 0.001), M stage (*P* < 0.001), histologic grade (*P* < 0.001), and surgery (*P* < 0.001) were independently and significantly associated with DSS (Table 2). All these independent prognostic factors for OS (0.5085) and DSS (0.5870) met the pH assumption as the Schönfeld residual test demonstrated (Figures 3(a) and 3(b)). Based on the above, all these independent prognostic factors were used to construct the nomograms to predict 3-, 5-, and 10-year OS and DSS (Figures 4(a) and 4(b)). For each patient who predicted survival rate, we drew a vertical line corresponding to each variable to calculate the specific point. Then the points of each variable were added, and the position where the vertical line intersected the survival axis on the total points' line was the survival rate of the patient.

### 3.4. Nomogram Validation

**Figure 5:**
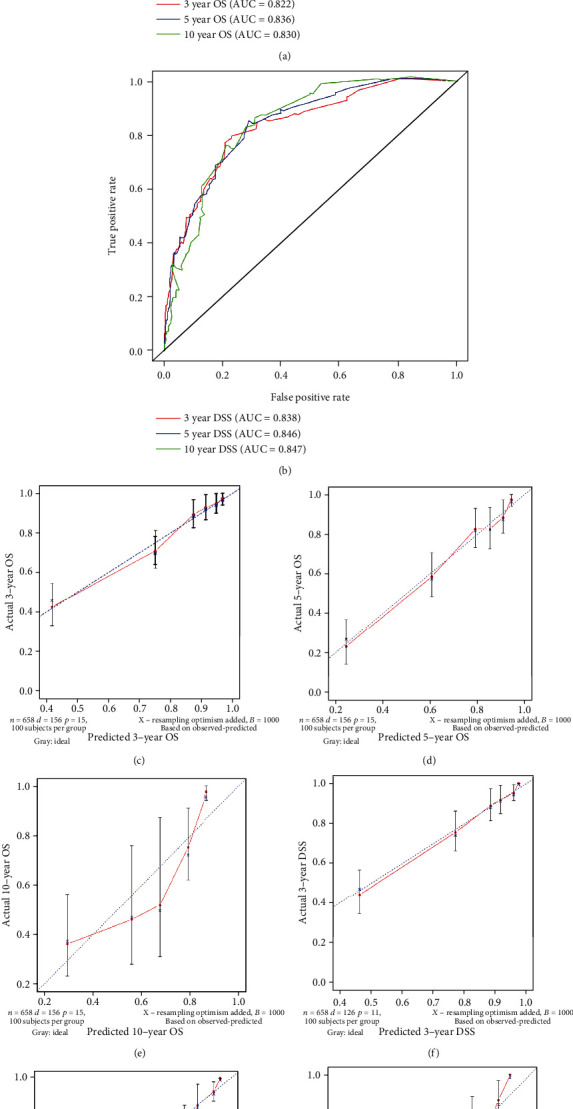
Validation of nomograms. (a) The ROC curves of the nomogram for predicting 3-, 5-, and 10-year OS; the area under curve was 0.822, 0.836, and 0.830, respectively. (b) The ROC curves of the nomogram for predicting 3-, 5-, 10-year DSS; the area under curve was 0.838, 0.846, and0.847, respectively. Calibration curves of the nomogram for predicting (c) 3-, (d) 5-, and (e) 10-year OS and the actual OS. Calibration curves of the nomogram for predicting (f) 3-, (g) 5-, and (h) 10-year DSS and the actual DSS.

**Figure 6:**
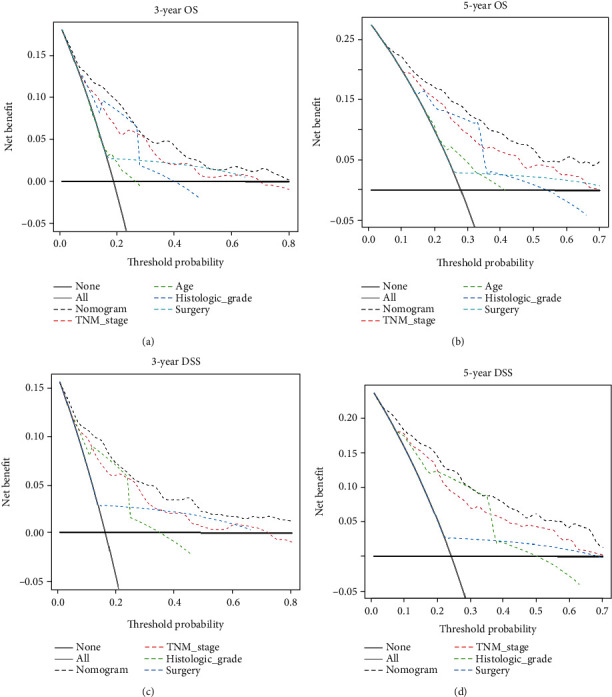
DCA curves of the nomograms and other independent prognostic factors. The DCA curves of the nomogram for predicting (a) 3- and (b) 5-year OS had a better net benefit compared to other independent prognostic factors. The DCA curves of the nomogram for predicting (c) 3- and (d) 5-year DSS showed better net benefit compared with other independent prognostic factors.

The performance of the nomogram was validated internally by 1,000 times bootstrap resampling. The *C*-indexes for the nomogram of 3-, 5-, and 10-year OS and DSS were 0.807 [95% confidence interval (CI), 0.772-0.841] and 0.836 (95% CI, 0.803-0.870), respectively. In 1,000 times bootstrap resampling, the *C*-indexes for the nomogram of 3-, 5-, and 10-year OS and DSS were 0.802 and 0.825, respectively, suggesting that these nomograms were accurate models for predicting OS and DSS. For the internal verification, the ROC showed that the nomograms for 3-, 5-, and 10-year OS both had a fairly good discriminatory ability (Figure 5(a)) with the AUC of 0.822, 0.836, and 0.830, respectively. For the nomograms of 3-, 5-, and 10-year DSS, the AUC were 0.838, 0.846, and 0.847, respectively (Figure 5(b)), also suggesting a fairly good discriminatory ability. The calibration curves based on 1,000 times bootstrap resampling indicated excellent consistency between the predicted and actual survival outcomes in the nomograms for predicting 3-, 5-, and 10-year OS and DSS (Figures 5(c)–5(h)). The DCA curves demonstrated that the nomogram of 3- and 5-year OS and DSS made favourable predictions and were superior to the TNM stage and other variables (Figures 6(a)–6(d)). Overall, the predictive nomograms were clinically useful and could make reasonable predictions.

## 4. Discussion

SACC is a malignant tumor with a seemingly benign histological appearance, characterized by indolent, locally invasive growth, and a high propensity for local recurrence and distant metastasis [20]. Complications of local recurrence and distant metastasis may lead to death in SACC patients. Therefore, it is necessary to establish a prognostic prediction model specifically designed for individual SACC patients. In this research, including 658 cases from the SEER∗Stat Database, age, T stage, N stage, M stage, histologic grade, and surgery were identified as independent prognostic factors for OS, and similarly, T stage, N stage, M stage, histologic grade, and surgery were identified as independent prognostic factors for DSS. Based on the above, nomograms were established and validated to effectively and visually predict the 3-, 5-, and 10-year OS and DSS of patients with SACC, and it performed well in predicting the survival of patients with many cancers [21]. With such predictive models, we may accurately predict the OS and DSS of patient with SACC easily with his personalized clinical parameters. Clinicians can calculate the total point of the SACC patient based on the nomograms, determine the patient's risk, and improve the treatment plan to obtain a better prognosis.

Adenoid cystic carcinoma is a rare tumor of the salivary glands, and the mainstay treatment modality of patient with SACC is surgery [22, 23]. In our study, we affirmed the impact of surgery on survival. The OS [HR = 0.384, 95% CI (0.221-0.668), *P* < 0.001] and DSS [HR = 0.283, 95% CI (0.156-0.511), *P* < 0.001] of SACC patients undergoing surgery were significantly increased. Elective neck dissection (END) and adjuvant radiotherapy are often applied after the initial surgery in cases with clinically evident metastasis. Cervical lymph node metastasis is rare in SACC and wide discrepancies in the incidence of lymph node metastasis reported by some authors, ranging from 4% to 33% [23–27]. Some studies reported that incidence of clinically positive nodes is low for SACC of the parotid gland and hard palate, but high for base of the tongue [28–30]. As seen in different origin sites of tumor, SACC may undergo neck metastasis via direct extension and lymphovascular spread [31]. For SACC patients with clinically negative lymph node status, it is still controversial on a potential advantage in reducing local and distant recurrence to improve survival rate by performing END. Interestingly, we found that the OS [HR = 0.997, 95% CI (0.585-1.701), *P* = 0.992] and DSS [HR = 1.094, 95% CI (0.621-1.927), *P* = 0.756] of SACC patients in N1 stage were not statistically different from those with negative lymph node status, while the OS [HR = 1.993, 95% CI (1.282-3.096), *P* = 0.002] and DSS [HR = 2.092, 95% CI (1.306-3.351), *P* = 0.002] of patients in N2 or N3 stages were significantly lower. Combined with the low incidence of cervical lymph node metastasis in SACC patients, and the 5-year OS of patients who received END was only 2.1% higher than that of non-received patients (Table 1), we believe that the clinical benefit of END for patients with negative lymph node status is poor. However, since the relative rarity of SACC, most reports of outcomes comprised all histologic types and small patient cohorts over extended periods in different locations of the head and neck, making it hard to develop definitive conclusions about treatment. Of note, cervical metastasis was less likely than distant metastasis, and the incidence was reported from 35% to 50% [31]. Our multivariate Cox proportional hazards model analysis also showed that distant metastasis is an independent prognostic factor of OS and DSS in SACC patients. The most accepted route of distant metastasis is hematogenous spread and would likely occur in the lungs. Therefore, routine chest radiographs for patients with adenoid cystic carcinoma are crucial [1, 32].

Surgery to completely remove the tumor is the gold standard for SACC treatment. Due to the delicate and complex anatomic structures of primary origin, the high risk of PNI, the surgical margins are difficult to be clear, and postoperative adjuvant radiotherapy is often required. [23, 33]. In our study, the 5-year OS of patients who received postoperative radiotherapy and those who did not receive were 80.4% and 82.2%, respectively. van Weert et al. demonstrated that patients treated with surgery and adjuvant irradiation did not have a better prognosis compared to patients treated with surgery alone [34]. Some authors indicated that postoperative radiotherapy did not improve the local control rates of SACC [35]. The role of postoperative adjuvant radiotherapy remains controversial, and conventional radiotherapy is not recommended as a single modality primary treatment [36]. However, for patients unfit for surgery or with inoperable disease, radiotherapy should be considered. Several studies showed that postoperative adjuvant radiotherapy is positively correlated with local control rate [37–40] and recommended postoperative radiotherapy for patients with positive microscopic margins and advanced T stage [41]. Due to the suboptimal outcome that many patients treated with surgery and adjuvant radiotherapy still experience local failures in a long run, a study postulates that postoperative radiotherapy likely delays rather than prevents tumor recurrences [42]. In order to improve the treatment outcome, other forms of irradiation, such as particularly neutron irradiation, were applied to locally advanced SACC patients and demonstrated as an effective therapy [43]. To our opinions, postoperative radiotherapy may improve the local control rates, but the impact on the survival rates of SACC patients still needs more research to prove. As the long-term prognosis is inferior and distant metastasis and late local recurrences may still occur at 10 to 15 years from diagnosis, long-term follow-up is required for these patients with SACC.

At present, patients' TNM stage is mostly used in clinical practice as the basis for formulating treatment plans and predicting prognosis. However, due to the complexity and heterogeneity of the occurrence and development of tumors, the current TNM staging system is too simple to fully consider other prognostic factors. Some studies have proved that the nomograms are scientific and precise, which can make it an alternative to the traditional TNM staging system [44, 45]. In this study, the DCA curves exhibited that the 3-year and 5-year OS and DSS nomograms had better clinical benefits than TNM stage, which means better clinical guidance.

In 2015, Lan et al. established a large-scale multiagency international data set on head and neck ACC patient, which combined the predictive factors of interest, including age, gender, tumor site, clinical T stage, perineural invasion, margin status, pathologic N-stage, and M-stage. In Lan et al.'s study, nomograms were constructed to predict 10-year recurrence-free probability, distant recurrence free probability, overall survival, and cancer-specific mortality of ACC patient and were further validated using external data sets of 99 patients from 2 other institutions [46]. Likewise, in 2017, Shen et al. constructed a cause-specific mortality prediction model for age, tumor size, advanced T stage, positive lymph node, metastasis, and surgery in patients with head and neck ACC based on the SEER database. [47]. Recently, based on the SEER database, Mu et al. performed the multivariate Cox proportional hazards model to screen out age, primary site, lymph node metastasis, distant metastasis, radiotherapy, and surgery as independent prognostic factors for DSS of ACC patients at different anatomical sites. Furthermore, a nomogram was constructed for predicting DSS, and external verification was carried out using the Chinese cohorts [44]. Compared with other predictive models, we used LASSO Cox regression analysis to evaluate the prognostic variables associated with SACC to construct a more simplified prediction model. In this study, the multivariate Cox proportional hazards model satisfying the pH assumption verified that these variables were independent prognostic factors and were used to construct nomograms to predict 3-, 5-, and 10-year OS and DSS in SACC patients. The DCA curves demonstrated that the nomograms of OS and DSS had good clinical benefit and were superior to the TNM stage and other variables in the prognostic prediction of SACC patients.

This study provides valuable information about the value of the nomogram for predicting the survival of patients with SACC, but it has several inherent limitations that need to be addressed. Firstly, our study is limited by the retrospective nature with some inevitable bias. Prospective randomized clinical trials or multicenter retrospective validation studies are needed to validate our results and improve the specificity and sensitivity of the nomogram for future clinical applications. Secondly, certain information which may have a great impact on survival of SACC patients was not available from the SEER registry, such as the molecular markers, perineural invasion (PNI), drinking, smoking, and positive surgical margins.

## 5. Conclusions

In conclusion, our study indicated that age, T stage, N stage, M stage, histologic grade, and surgery were identified as independent prognostic factors for OS, and similarly, T stage, N stage, M stage, histologic grade, and surgery were identified as independent prognostic factors for DSS in patients with SACC. Meanwhile, we successfully constructed and carefully evaluated nomograms that provided satisfactory accuracy for predicting the 3- and 5-year OS and DSS in such patients with SACC. These nomograms may be helpful for providing a prognostic reference and optimizing individual therapies and follow-up for other SACC patients.

## Figures and Tables

**Table 1:** Characteristics and survival rate of patients with salivary gland adenoid cystic carcinoma.

Number of patients (*n* = 658) (%)			Overall survival (%)				Disease-specific survival (%)			
Characteristic	Frequency	Percent (%)	3-year	5-year	10-year	Last follow-up	3-year	5-year	10-year	Last follow-up
Age										
<40 years	116	17.6	90.5	88.8	85.3	85.3	90.5	88.8	85.3	85.3
40-49 years	116	17.6	87.1	81.0	78.6	77.6	89.7	83.6	79.3	79.3
50-59 years	138	21.0	89.1	86.2	83.3	82.6	89.1	86.2	84.1	84.1
60-69 years	136	20.7	82.4	77.2	73.8	72.8	86.8	83.1	80.1	80.1
≥70 years	152	23.1	79.6	73.0	67.1	65.8	83.6	78.3	76.3	75.7
Gender										
Female	379	57.6	86.0	81.5	77.6	77.0	87.6	83.6	81.3	81.0
Male	279	42.4	84.6	79.9	75.6	75.3	87.8	83.9	80.3	80.3
Race										
White	503	76.4	86.1	81.1	76.7	76.3	88.7	84.5	81.7	81.7
Black	71	10.8	81.7	81.7	76.1	76.1	84.5	84.5	78.9	78.9
Other	84	12.8	84.5	78.6	77.4	76.2	84.5	78.6	77.4	76.2
Marital status										
Married	380	57.7	85.8	81.6	77.1	76.6	88.2	84.7	80.8	80.5
Single	129	19.6	86.8	82.9	79.8	79.1	88.4	85.3	82.2	82.2
Separated	11	1.7	81.8	72.7	72.7	72.7	81.8	72.7	72.7	72.7
Divorced	58	8.8	87.9	86.2	81.0	81.0	89.7	87.9	87.9	87.9
Widowed	53	8.1	77.4	73.6	67.9	67.9	81.1	77.4	77.4	77.4
Unknown	27	4.1	85.2	66.7	66.7	66.7	88.9	70.4	70.4	70.4
Primary site of tumor										
Parotid gland	199	30.2	87.4	82.9	79.9	79.9	88.4	84.4	82.4	82.4
Palate	104	15.8	89.4	85.6	82.7	81.7	93.3	91.3	91.3	90.4
Other major salivary glands	219	33.3	84.0	77.6	73,5	72.6	85.8	79.9	76.7	76.7
Other minor salivary glands	136	20.7	81.6	79.4	72.8	72.8	85.3	83.1	77.2	77.2
AJCC stage										
I	171	26.0	96.5	94.7	91.2	90.1	97.7	96.5	94.7	94.2
II	150	22.8	94.7	90.7	86.0	84.7	96.7	94.0	91.3	91.3
III	119	18.1	86.6	82.4	78.2	78.2	89.1	84.9	80.7	80.7
IVA	139	21.1	77.0	69.8	64.0	64.0	80.6	73.4	69.1	69.1
IVB	29	4.4	58.6	48.3	48.3	48.3	65.5	58.6	58.6	58.6
IVC	50	7.6	56.0	50.0	48.0	48.0	56.0	50.0	48.0	48.0
T stage										
T1	190	28.9	92.6	90.5	87.4	86.8	93.7	92.1	90.5	90.0
T2	179	27.2	93.9	89.9	86.0	84.9	95.5	92.7	90.5	90.5
T3	126	19.1	78.6	72.2	66.7	66.7	81.7	75.4	69.0	69.0
T4a	132	20.1	77.3	71.2	65.1	65.9	80.3	74.2	71.2	71.2
T4b	31	4.7	54.8	45.2	45.2	45.2	61.3	54.8	54.8	54.8
N stage										
N0	533	81.0	89.5	84.8	80.9	80.3	91.9	88.0	85.4	85.2
N1	70	10.6	80.0	78.6	74.3	74.3	80.0	78.6	75.7	75.7
N2a	4	0.6	50.0	50.0	50.0	50.0	50.0	50.0	50.0	50.0
N2b	44	6.7	52.3	43.2	40.9	40.9	56.8	47.7	43.2	43.2
N2c+N3	7	1.1	57.1	57.1	28.6	28.6	57.1	57.1	42.9	42.9
M stage										
M0	608	92.4	87.8	83.4	79.1	78.6	90.3	86.5	83.6	83.4
M1	50	7.6	56.0	50.0	48.0	48.0	56.0	50.0	48.0	48.0
Grade										
Low-grade	161	24.5	95.7	94.4	90.7	90.1	96.3	95.7	93.2	92.5
Intermediate-grade	309	46.9	92.6	88.3	84.1	83.5	94.1	90.9	88.3	88.3
High-grade	113	17.2	63.7	55.8	54.0	54.0	68.1	61.1	58.4	58.4
High-grade transformation	75	11.4	66.7	58.7	50.7	50.7	72.0	62.7	57.3	57.3
Surgery										
No	29	4.4	44.8	41.4	34.5	34.5	44.8	44.8	37.9	37.9
Yes	629	95.6	87.3	82.7	82.7	78.2	89.7	85.5	82.8	82.7
Radiation										
No	169	25.7	87.6	82.2	76.9	76.3	91.7	87.0	84.6	84.0
Yes	489	74.3	84.7	80.4	76.7	76.3	86.3	82.6	79.6	79.6
Chemotherapy										
No	586	89.1	88.2	83.6	79.4	78.8	90.4	86.5	83.8	83.6
Yes	72	10.9	62.5	58.3	55.6	55.6	65.3	61.1	56.9	56.9
Neck dissection										
No	295	44.8	86.8	82.0	79.3	79.0	88.8	84.7	83.7	83.4
Yes	363	55.2	84.3	79.9	74.7	74.1	86.8	82.9	78.5	78.5

**Table 2:** Multivariate cox regression analysis of various factors associated with overall and disease specific survival in patients with salivary gland adenoid cystic carcinoma.

Characteristic	OS		DSS	
	HR (95% CI)	*P*	HR (95% CI)	*P*
Age		<0.001		
<40 years	Reference			
40-49 years	1.153 (0.617-2.155)	0.655		
50-59 years	0.930 (0.494-1.751)	0.823		
60-69 years	1.632 (0.906-2.939)	0.103		
≥70 years	2.377 (1.342-4.211)	0.003		
T stage		<0.001		<0.001
T1	Reference		Reference	
T2	1.160 (0.667-2.016)	0.599	0.880 (0.452-1.715)	0.708
T3	2.429 (1.445-4.085)	<0.001	2.493 (1.399-4.444)	0.002
T4a	2.420 (1.441-4.064)	<0.001	2.609 (1.452-4.687)	0.001
T4b	3.388 (1.794-6.400)	<0.001	3.198 (1.575-6.495)	0.001
N stage		0.001		0.001
N0	Reference		Reference	
N1	0.997 (0.585-1.701)	0.992	1.094 (0.621-1.927)	0.756
N2+N3	1.993 (1.282-3.096)	0.002	2.092 (1.306-3.351)	0.002
M stage		<0.001		<0.001
M0	Reference		Reference	
M1	3.084 (1.871-5.083)	<0.001	3.124 (1.885-5.178)	<0.001
Histologic grade		<0.001		<0.001
Low-grade transformation	Reference		Reference	
Intermediate-grade transformation	1.847 (1.040-3.282)	0.036	1.656 (0.852-3.220)	0.137
High-grade transformation	5.081 (2.816-9.168)	<0.001	5.983 (3.074-11.643)	<0.001
High-grade transformation	5.104 (2.789-9.341)	<0.001	5.723 (2.8860 11.3490)	<0.001
Surgery		<0.001		<0.001
No	Reference		Reference	
Yes	0.384 (0.221-0.668)	<0.001	0.283 (0.156-0.511)	<0.001

## Data Availability

The data analyzed during the current study are available from the SEER data set repository.

## Additional Points

*Reporting Checklist*. The authors have completed the TRIPOD reporting checklist.

## Supplementary Materials

Supplementary Materials Table S1: coefficients of each predictor returned by Cox regression.
