# Supplementary material for: Clinical Prediction Nomograms to Assess Overall Survival and Disease-Specific Survival of Patients with Salivary Gland Adenoid Cystic Carcinoma
Source: Biomed Res Int. 2022 Aug 29;2022:7894523. doi: 10.1155/2022/7894523 (PMC9444442; doi:10.1155/2022/7894523)
Supplement: Supplementary Materials — Table S1: coefficients of each predictor returned by Cox regression. [file 7894523.f1.zip › Table S1.docx]

**Table S1.** **Coefficients of each predictor returned by Cox regression**

| Characteristic | exp(coef) | exp(-coef) | lower 95%CI | upper 95%CI |
| --- | --- | --- | --- | --- |
| OS |  |  |  |  |
| Age | 1.2714 | 0.7865 | 1.1243 | 1.4379 |
| Histologic grade | 1.7887 | 0.5591 | 1.5181 | 2.1076 |
| T stage | 1.3913 | 0.7187 | 1.2196 | 1.5872 |
| N stage | 1.4367 | 0.696 | 1.1552 | 1.7869 |
| M stage | 2.7764 | 0.3602 | 1.7232 | 4.4732 |
| Surgery | 0.4418 | 2.2633 | 0.2597 | 0.7516 |
| DSS |  |  |  |  |
| Histologic grade | 1.897 | 0.527 | 1.5765 | 2.2836 |
| T stage | 1.449 | 0.6899 | 1.2495 | 1.6814 |
| N stage | 1.553 | 0.6437 | 1.2283 | 1.9646 |
| M stage | 3.085 | 0.3241 | 1.8714 | 5.0865 |
| Surgery | 0.373 | 2.6806 | 0.2101 | 0.6623 |
